# Inflammatory Mediator TAK1 Regulates Hair Follicle Morphogenesis and Anagen Induction Shown by Using Keratinocyte-Specific TAK1-Deficient Mice

**DOI:** 10.1371/journal.pone.0011275

**Published:** 2010-06-23

**Authors:** Koji Sayama, Kentaro Kajiya, Koji Sugawara, Shintaro Sato, Satoshi Hirakawa, Yuji Shirakata, Yasushi Hanakawa, Xiuju Dai, Yumiko Ishimatsu-Tsuji, Daniel Metzger, Pierre Chambon, Shizuo Akira, Ralf Paus, Jiro Kishimoto, Koji Hashimoto

**Affiliations:** 1 Department of Dermatology, Ehime University Graduate School of Medicine, Ehime, Japan; 2 Skin Biology Research Group, Shiseido Innovative Science Research and Development Center, Yokohama, Japan; 3 Department of Dermatology, University of Luebeck, Luebeck, Germany; 4 Department of Microbiology and Immunology, The Institute of Medical Science, The University of Tokyo, Tokyo, Japan; 5 Institut de Génétique et de Biologie Moléculaire et Cellulaire (IGBMC), CNRS, INSERM, UdS, Collège de France, Illkirch, France; 6 Research Institute for Microbial Disease, Osaka University, Suita, Japan; 7 School of Translational Medicine, University of Manchester, Manchester, United Kingdom; Ecole Normale Supérieure de Lyon, France

## Abstract

Transforming growth factor-β-activated kinase 1 (TAK1) is a member of the NF-κB pathway and regulates inflammatory responses. We previously showed that TAK1 also regulates keratinocyte growth, differentiation, and apoptosis. However, it is unknown whether TAK1 has any role in epithelial–mesenchymal interactions. To examine this possibility, we studied the role of TAK1 in mouse hair follicle development and cycling as an instructive model system. By comparing keratinocyte-specific TAK1-deficient mice (*Map3k7* ^fl/fl^K5-Cre) with control mice, we found that the number of hair germs (hair follicles precursors) in *Map3k7* ^fl/fl^K5-Cre mice was significantly reduced at E15.5, and that subsequent hair follicle morphogenesis was retarded. Next, we analyzed the role of TAK1 in the cyclic remodeling in follicles by analyzing hair cycle progression in mice with a tamoxifen-inducible keratinocyte-specific TAK1 deficiency (*Map3k7* ^fl/fl^K14-Cre-ER^T2^). After active hair growth (anagen) was induced by depilation, TAK1 was deleted by topical tamoxifen application. This resulted in significantly retarded anagen development in TAK1-deficient mice. Deletion of TAK1 in hair follicles that were already in anagen induced premature, apoptosis-driven hair follicle regression, along with hair follicle damage. These studies provide the first evidence that the inflammatory mediator TAK1 regulates hair follicle induction and morphogenesis, and is required for anagen induction and anagen maintenance.

## Introduction

The NF-κB pathway mediates innate immune or pro-inflammatory responses, such as signaling by Toll-like receptors (TLRs), the IL-1 receptor (IL-1R), and tumor necrosis factor receptor (TNFR) [1], [2]. Transforming growth factor-β-activated kinase 1 (TAK1) is a member of the MAP3 kinase family [3] and an important member of the NK-κB pathway, involved in IL-1 and TNF-α-induced activation of NF-κB and MAP kinases [1]. Upon ligand binding, TNF-receptor-associated factor (TRAF) 6 or TRAF2 [4], [5], [6] activates TAK1, which then phosphorylates IκB kinases (IKKs), resulting in NF-κB activation.

Because members of the NF-κB pathway are increasingly recognized as important in the regulation of epithelial–mesenchymal interaction systems, ranging from tooth development to hair follicle induction and morphogenesis [7], [8], [9], [10], [11], we were interested in learning whether TAK1 also played a role in the biology of the hair follicle, a prototypic epithelial–mesenchymal interaction system. This interest was further fueled by our previous discovery in keratinocyte-specific TAK1-deficient mice (*Map3k7* ^fl/fl^K5-Cre) that TAK1 regulates keratinocyte growth, differentiation, and apoptosis [12]. However, the role of TAK1 in hair follicles has not been previously studied.

Induction and morphogenesis of the hair follicle [13] is controlled by complex signaling networks within the skin epithelium and between the epithelium and specialized inductive fibroblasts in its adjacent mesenchyme [7], [11]. Among these signaling networks, the NF-κB pathway and Wnt/β-catenin signaling provide central controls [7], [8]; however, the exact relationship between these signaling networks is not fully understood. Binding of EdaA1 to its receptor EdaR in the embryo is essential for the development of ectodermal appendages [14], and mutations in these genes cause reduced or absent ectodermal appendages [15], [16], [17], [18], [19]. Subsequently, the EdaA1/EdaR pathway activates the downstream NF-κB pathway [20]. A recent report showed that Wnt/β-catenin signaling lies both upstream and downstream of the EdaR/NF-κB pathway [8]. Wnt/β-catenin signaling within the epithelial cells is required for activation of the Eda/EdaR/NF-κB pathway at an early stage of hair follicle development [8], and the expression of Eda and EdaR requires Wnt/β-catenin signaling [8]. At a later stage, maintenance of Wnt signaling and elevated Wnt10a, Wnt10b, and Dkk4 expression requires the Eda/EdaR/NF-κB pathway [8].

The postnatal hair cycle in mice begins with catagen induction around P17, followed by the first telogen. Recently, the EdaR pathway has been shown to be involved in the hair cycle [9], [21], [22]. The expression of EdaA1 and EdaR increases in the anagen-catagen phase [21]. Furthermore, EdaA1 prolongs the anagen phase [22]. Thus, in addition to its well-established role in hair follicle morphogenesis, the EdaR pathway is also involved in hair cycle control.

Since TAK1 is a member of NF-κB pathway, we hypothesized that TAK1 is involved in hair follicle morphogenesis and hair cycle control. To explore this, we studied hair follicle development in keratinocyte-specific TAK1-deficient (*Map3k7* ^fl/fl^K5-Cre) mice and subsequent hair follicle cycling in tamoxifen-inducible keratinocyte-specific TAK1 deficient mice (*Map3k7* ^fl/fl^K14-Cre-ER^T2^) to avoid gene-targeting in embryonic development because this might damage the hair follicle, impairing its later capacity to cycle. These studies provide the first evidence that TAK1 regulates hair follicle induction, morphogenesis, and cycling.

## Materials and Methods

### Ethics Statement

The protocol for generating *Map3k7* ^fl/fl^K5-Cre mice and *Map3k7* ^fl/fl^K14-Cre-ER^T2^ mice was approved by the Institutional Review Board of Ehime University Graduate School of Medicine (#I-20-13 and #NE-27-16).

### Generation of keratinocyte-specific TAK1-deficient mice (*Map3k7* ^fl/fl^ K5-Cre)

TAK1 is encoded by the *Map3k7* gene. The targeting construct has been described previously [1]. We generated keratinocyte-specific TAK1-deficient mice (*Map3k7* ^fl/fl^K5-Cre) by breeding *Map3k7* ^fl/fl^ mice (C57Bl/6 background) with K5-Cre mice (C57Bl/6 background) [23], as previously described [12].

### Generation of tamoxifen-inducible keratinocyte-specific TAK1-deficient mice (*Map3k7* ^fl/fl^ K14-Cre-ER^T2^)

*Map3k7K* ^fl/fl^ mice were bred with K14-Cre-ER^T2^ mice (C57Bl/6 background) [24], [25] to generate *Map3k7* ^fl/fl^K14-Cre-ER^T2^ mice. We applied 100 µL of 4-hydroxytamoxifen (Sigma-Aldrich Co., St. Louis, MO) in ethanol at a concentration of 1 mg/mL topically to the dorsal skin of 8-week-old female *Map3k7* ^fl/fl^K14-Cre-ER^T2^ mice for 5 consecutive days [24].

### Wax depilation

The hair cycle was synchronized in the dorsal skin of 8-week-old female mice by wax (SURGI-WAX™, Ardell International, Los Angeles, CA) depilation, as described previously [26].

### Histological analysis

The stages of hair follicle morphogenesis, cycling, and dystrophic catagen were morphologically defined using dorsal skin of the mice and the score was defined as follows.

The hair morphogenesis stage of each hair follicle in each mouse group was evaluated as described previously [13]. At least 40 hair follicles or all hair follicles in the mice were evaluated in each mouse group (two mice/group). The score of each hair morphogenesis stage was defined as follows: stage 1 = 1, stage 2 = 2, stage 3 = 3, stage 4 = 4, stage 5 = 5, stage 6 = 6, stage 7 = 7, and stage 8 = 8. Then, the rate (%) of a certain hair morphogenesis stage in the total hair follicles and the median score in each mouse group were determined. The score of *Map3k7* ^fl/fl^K5-Cre mice was compared with *Map3k7* ^fl/fl^ mice. Statistical significance was determined using a Mann-Whitney's U-test. A difference of *P<0.01 was considered statistically significant.

The hair cycle stage of each hair follicle in each mouse group was evaluated as described previously [27]. At least 40 hair follicles were evaluated in each mouse group (two mice/group). The score of each hair cycle stage was defined as follows: catagen I = 1, catagen II = 2, catagen III = 3, catagen IV = 4, catagen V = 5, catagen VI = 6, catagen VII = 7, catagen VIII = 8, telogen  = 9, anagen I = 10, anagen II = 11, anagen IIIa = 12, anagen IIIb = 13, anagen IIIc = 14, anagen IV = 15, anagen V = 16, and anagen VI = 17. Then, the rate (%) of a certain hair cycle stage in the total hair follicles and the median score in each mouse group were determined. The score of tamoxifen-treated *Map3k7* ^fl/fl^K14-Cre-ER^T2^ mice was compared with the control mice. Statistical significance was determined using a Mann-Whitney's U-test. A difference of *P<0.01 was considered statistically significant.

Dystrophic catagen was defined according to a previous report [28] as early dystrophic catagen, mid dystrophic catagen, late dystrophic catagen, and dystrophic telogen. Then, the rate (%) of a certain hair follicle stage per total hair follicles was calculated in each mouse group. At least 40 hair follicles were evaluated in each mouse group (two mice/group).

## Results

### Impaired hair follicle morphogenesis in keratinocyte-specific TAK1-deficient mice

**Figure 1:**
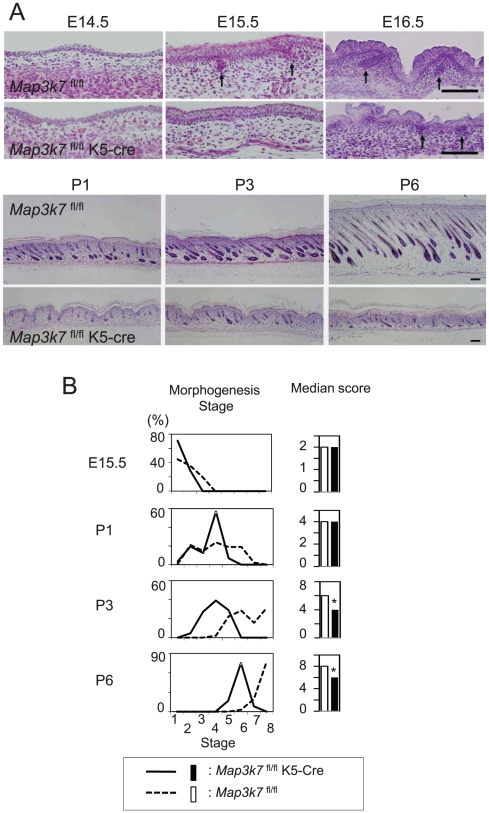
Impaired hair follicle morphogenesis in keratinocyte-specific TAK1-deficient mice. (A) Histological analysis of hair follicle development from E14.5 to P6. *Map3k7* ^fl/fl^K5-Cre were keratinocyte-specific TAK1-deficient mice [12]. *Map3k7* ^fl/fl^ mice were used as controls. Arrows indicate hair germs or hair pegs. Scale bar, 100 µm. (B) The hair morphogenesis stage of each hair follicle in each mouse group was evaluated as described previously [13]. Then, the rate (%) of a certain hair morphogenesis stage in the total hair follicles (left panel) and the median score in each mouse group (right panel) were determined. The score of *Map3k7* ^fl/fl^K5-Cre mice was compared with *Map3k7* ^fl/fl^ mice. Statistical significance was determined using a Mann-Whitney's U-test. *P<0.01.

Keratinocyte-specific TAK1-deficient (*Map3k7* ^fl/fl^K5-Cre) mice were generated, as previously described [12]. *Map3k7* ^fl/fl^ mice were used as controls. Histological analysis of hair follicle development is shown in Figure 1A. Although hair germs (follicle precursors) and dermal condensations appeared in both types of mice at E15.5, the number of hair germs in *Map3k7* ^fl/fl^K5-Cre mice was significantly lower than that in *Map3k7* ^fl/fl^ mice (Fig. 1A). At E16.5, the hair germ further progressed into the hair peg stage of hair follicle morphogenesis in *Map3k7* ^fl/fl^ mice at the rate expected for wild-type mice [29], while hair pegs were essentially absent in *Map3k7* ^fl/fl^K5-Cre mice. Similarly, at P1-6, postnatal hair follicle development was severely impaired in *Map3k7* ^fl/fl^K5-Cre mice (Fig. 1A).

Hair follicle morphogenesis was quantitatively analyzed. The morphogenesis stage and the median score of each mouse group are shown in Fig. 1B. Hair follicle morphogenesis indicators were significantly delayed in *Map3k7* ^fl/fl^K5-Cre mice, as evident from their lower hair morphogenesis score, compared with *Map3k7* ^fl/fl^ mice at P3 and P6 (Fig. 1B).

### Generation of tamoxifen-inducible keratinocyte-specific TAK1-deficient mice

**Figure 2:**
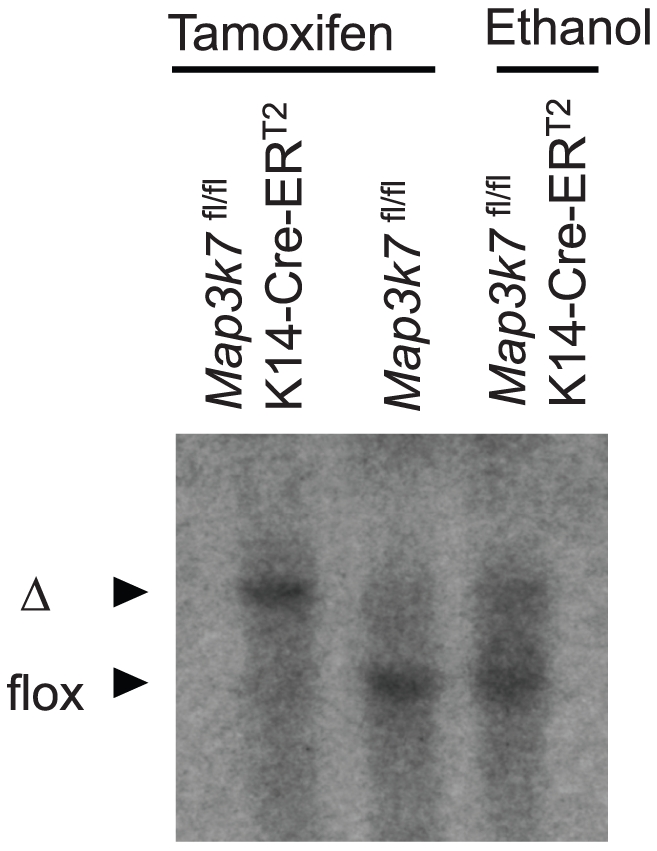
Southern blot analysis. Genomic DNA prepared from the ear skin of mice treated with tamoxifen solution (15 µL/ear) or ethanol for 5 consecutive days was digested with *Xba*I and *Eco*RI. Southern blot analysis for the deletion of the floxed *Tak1* allele was performed as described previously [1]. Cre expression resulted in excision of the floxed allele (*flox*) and generated the deleted allele (Δ) of *Map3k7*.

Because germline targeting of TAK1 greatly disrupted hair morphogenesis and, thus, precluded a meaningful analysis of subsequent hair follicle cycling, we next used tamoxifen-inducible keratinocyte-specific TAK1-deficient mice (*Map3k7* ^fl/fl^K14-Cre-ER^T2^), in which Cre-ER^T2^ was expressed in the epidermis under the control of the K14 promoter [24]. Southern blot analysis demonstrated efficient deletion of the floxed allele in the epidermis of tamoxifen-treated *Map3k7* ^fl/fl^K14-Cre-ER^T2^ mice (Fig. 2).

Although, the skin sample for Southern blot analysis contained non-keratinocyte cells, such as Langerhans cells, melanocytes, or fibroblasts, the band of tamoxifen-treated *Map3k7* ^fl/fl^K14-Cre-ER^T2^ mice was a single-recombined band. Since, the majority of this skin sample consists of keratinocytes, flox band of non-keratinocyte cells may not be apparent in this blot. Ethanol-treated *Map3k7* ^fl/fl^K14-Cre-ER^T2^ mice also show a minor recombination band, presumably due to slightly leaky Cre- ER^T2^ activity.

### Keratinocyte-specific TAK1 deletion results in hair loss in adolescent mice

**Figure 3:**
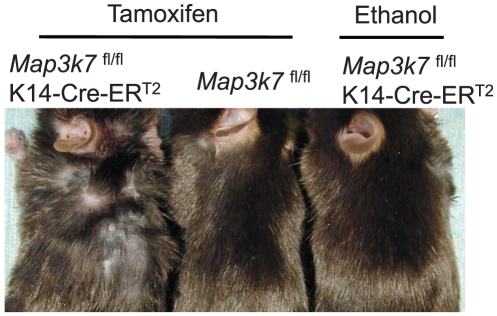
Keratinocyte-specific TAK1 deletion results in hair loss in adolescent mice. Tamoxifen was topically applied to the dorsal skin of the *Map3k7* ^fl/fl^K14-Cre-ER^T2^ mice for 5 consecutive days to delete TAK1. The clinical appearance of the mice 4 weeks after the application is shown. As controls, *Map3k7* ^fl/fl^ mice or *Map3k7* ^fl/fl^K14-Cre-ER^T2^ mice were treated with tamoxifen or ethanol, respectively.

In the first experiment, tamoxifen was simply applied to the dorsal skin of 8 weeks old mice for 5 consecutive days. Two weeks after the application, the tamoxifen-treated *Map3k7* ^fl/fl^K14-Cre-ER^T2^ mice started to lose their hair shafts, and this process continued for more than 4 weeks (Fig. 3). This phenotype suggested the involvement of TAK1 in hair follicle cycling.

### Keratinocyte-specific TAK1 deletion delays hair cycle progression in adolescent mice

**Figure 4:**
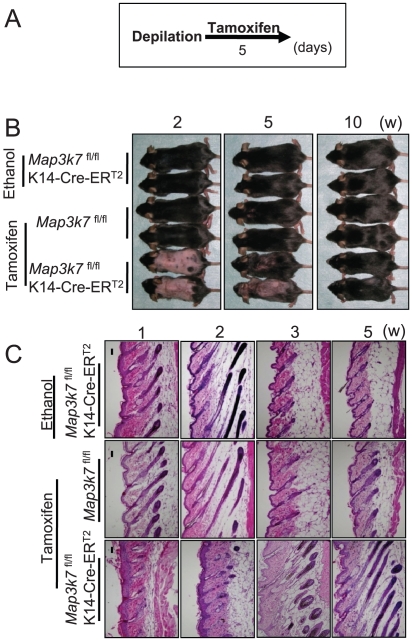
Keratinocyte-specific TAK1 deletion delays hair cycle progression in adolescent mice. (A) Schedule of tamoxifen application. The hair cycle was synchronized to anagen phase by wax depilation, and tamoxifen was topically applied to the dorsal skin of *Map3k7* ^fl/fl^K14-Cre-ER^T2^mice to delete TAK1. As controls, *Map3k7* ^fl/fl^ mice or *Map3k7* ^fl/fl^K14-Cre-ER^T2^ mice were treated with tamoxifen or ethanol, respectively. (B) Clinical appearance at the indicated time point after the depilation. At 2 weeks, hair shaft formation was noted in the control mice, while hair shaft growth was not seen in the tamoxifen-treated *Map3k7* ^fl/fl^K14-Cre-ER^T2^ mice. (C) Histological analysis at the indicated time point after the depilation. Anagen progression was severely delayed in TAK1-deleted mice at 1–3 weeks. Scale bar, 100 µm.

To further dissect the role of TAK1 in hair follicle cycling, synchronized, active hair growth (anagen) was induced in resting (telogen) hair follicles by wax depilation [26]. This was followed by topical tamoxifen application to the dorsal skin, to delete TAK1 (Fig. 4A). At 2 weeks after synchronized anagen induction, hair shaft formation was noted in the control mice, as a macroscopic indicator of well-advanced anagen development, while hair shaft growth was not seen in the tamoxifen-treated *Map3k7* ^fl/fl^K14-Cre-ER^T2^ mice (Fig. 4B). Histological analysis revealed that anagen progression was severely delayed in TAK1-deleted mice at 1–3 weeks (Fig. 4C).

**Figure 5:**
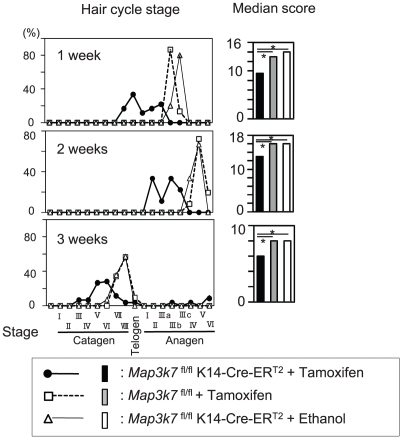
Quantitative hair cycle analysis. Hair cycle stage of each hair follicle in each mouse group in Fig. 4 was evaluated according to our previous report [27]. Then, the rate (%) of a certain hair cycle stage in the total hair follicles (left panel) and the median score in each mouse group (right panel) were determined. The score of tamoxifen-treated *Map3k7* ^fl/fl^K14-Cre-ER^T2^ mice was compared with tamoxifen-treated *Map3k7* ^fl/fl^ mice or ethanol-treated *Map3k7* ^fl/fl^K14-Cre-ER^T2^ mice. Statistical significance was determined using a Mann-Whitney's U-test. *P<0.01.

Quantitative hair cycle histomorphometry and hair cycle score calculation (Fig. 5) confirmed that depilation-induced anagen progression was severely delayed in TAK1-deleted mice at 1–3 weeks, while anagen development progressed as expected in TAK1-competent mice. Taken together, these data suggest that TAK1 is an important regulator of early anagen development in telogen hair follicles, although TAK1 does not appear to be indispensable for anagen induction.

Although leaky Cre- ER^T2^ activity was noted in ethanol-treated *Map3k7* ^fl/fl^K14-Cre-ER^T2^ mice (Fig. 2), there was no significant difference between ethanol-treated *Map3k7* ^fl/fl^K14-Cre-ER^T2^ mice and tamoxifen-treated *Map3k7* ^fl/fl^ mice. Therefore, leaky activity of Cre- ER^T2^ seems to have a minimum effect in this experiment.

### Keratinocyte-specific TAK1 deletion causes a transition from anagen to dystrophic catagen in adolescent mice

**Figure 6:**
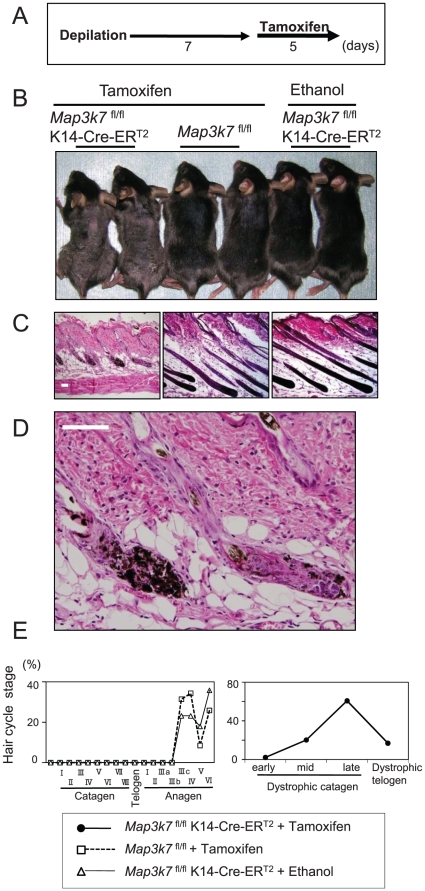
Keratinocyte-specific TAK1 deletion causes a transition from anagen to dystrophic catagen in adolescent mice. (A) Schedule of tamoxifen application. The hair cycle was synchronized to anagen phase by wax depilation. At 7 days after depilation, tamoxifen was applied for 5 days. (B) Clinical appearance of the mice 2 weeks after depilation. (C) Histological analysis of the mice 2 weeks after depilation. (D) Higher magnification of the tamoxifen-treated *Map3k7* ^fl/fl^K14-Cre-ER^T2^ mice in (C). Scale bar, 100 µm. (E) Dystrophic catagen was defined according to a previous report [28]. Then, the rate (%) of a certain hair follicle stage per total hair follicles was calculated. Quantitative analyses confirmed that most of the hair follicles in tamoxifen-treated *Map3k7* ^fl/fl^K14-Cre-ER^T2^ mice were in late dystrophic catagen, while those of controls were in anagen.

In the third experimental setup, TAK1 was deleted only 1 week after anagen induction (Fig. 6A). At 2 weeks after depilation, hair regrowth was reduced in the tamoxifen-treated *Map3k7* ^fl/fl^K14-Cre-ER^T2^ mice (Fig. 6B), compared with controls. Histological analysis revealed that almost all of the hair follicles in tamoxifen-treated *Map3k7* ^fl/fl^K14-Cre-ER^T2^ mice had prematurely entered the apoptosis-driven regression stage of hair follicle cycle (i.e., catagen; Fig. 6C).

Interestingly, however, this accelerated catagen development was associated with striking pigmentary signs of hair follicle damage (dystrophy): many large, ectopically located melanin clumps, often larger than keratinocyte nuclei, were found not only in their normal location (i.e., the precortical hair matrix), but also eccentrically in the hair bulb periphery and in the epithelial strand of the involuting catagen hair follicles (Fig. 6D). Thus, TAK1 deletion induced “dystrophic catagen,” an indicator of major hair follicle damage [28]. Quantitative analyses (Fig. 6E) confirmed that most of the hair follicles in tamoxifen-treated *Map3k7* ^fl/fl^K14-Cre-ER^T2^ mice were in late dystrophic catagen, while those of controls were in anagen. These data suggest that TAK1 is essential for maintaining a functional anagen phase.

## Discussion

Here, we show by mouse genomics and targeted deletion experiments that TAK1, a member of the NF-κB pathway that has chiefly been recognized as a mediator of innate and adaptive immunity [1], [2], [4], [5], [6], [30], is also a key component of the molecular machinery that controls murine hair growth. Consistent with our previous discovery that TAK1 regulates keratinocyte growth, differentiation, and apoptosis [12], we now show that the selective deletion of TAK1 in keratinocytes retards hair follicle induction, morphogenesis, and anagen development, and is required for the maintenance of normal anagen. This newly identified role of TAK1 in hair follicle development and cycling implicates TAK1 as a novel player in complex organ remodeling events and epithelial-mesenchymal interactions, which can be modeled by murine hair follicles [11].

The TAK1-NF-κB pathway regulates not only immune responses [1], [30], but also epithelial function [12]. Because the NF-κB pathway controls pro-inflammatory responses, deletion of this pathway was expected to suppress epithelial inflammation. Unexpectedly, however, deletion of TAK1, IKK-β, or IKK-γ was found to result in severe skin inflammation (including abscess formation) [12], [31], [32], [33]. Similarly, a lack of NF-κB signaling produced by the conditional ablation of IKKγ or IKKα and IKKβ in the intestinal epithelium caused severe chronic intestinal inflammation in mice [34]. This suggests that a continuous, basal level of NF-κB activation may be required to maintain epithelial integrity and homeostasis and to suppress excessive skin inflammation. In the current study, we add to the established role of TAK1 in murine skin the novel function of hair growth control.

Since TAK1 regulates keratinocyte function [12], there is a possibility that hair follicle defects in TAK1-deficient mice might be attributed to such abnormal capacities of keratinocytes rather than reflecting the specific function of TAK1 in hair follicle regulation. However, clinical skin pheno types and histological abnormalities were not apparent at birth and started to appear at P2 in *Map3k7* ^fl/fl^K5-Cre mice [12]. On the other hand, E15.5 is the time point when the defect of hair follicle development became apparent (Fig. 1). Therefore, the defect of hair follicle development is primarily due to the defect of TAK1 signaling during embryogenesis, rather than the functional defect of keratinocytes.

Recently, TAK1 binding protein (TAB) 2 has been identified as a binding partner of EdaR-associated death domain protein (EDARADD) using a yeast two-hybrid screening [35]. In 293 cells, endogenous and overexpressed TAB2, TRAF6 and TAK1 were co-immunoprecipitated with EDARADD [35]. Furthermore, dominant negative forms of TAB2, TRAF6 and TAK1 blocked the NF-κB activation induced by EDARADD in 293 cells[35]. Therefore, it is suggested that TAK1 is involved in hair follicle development. However, the actual role of TAK1 in hair follicle has not been studied before.

**Figure 7:**
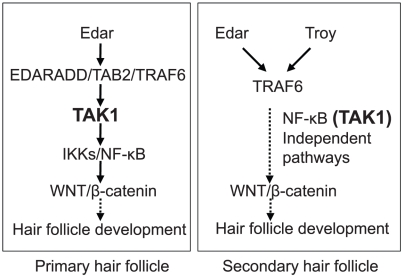
Model for the signaling pathways involved in hair follicle development. In the development of primary hair placodes, TAK1 is involved in the Edar/NF-κB pathway. In secondary hair placodes, NF-κB (TAK1)-independent pathways regulate hair placode development.

The coats of mice contain four major hair follicle subpopulations: guard hairs, awl and auchene hairs, and zigzag hairs. Formation of each kind of hair follicle starts at E14, E16, and E18-P3, respectively and the regulatory mechanisms of hair follicle development are slightly different among them [7], [36]. Epidermal NF-κB activity is first observed in the placodes of primary guard hairs at E14.5 [36]. In the absence of NF-κB activity, downstream events, such as maintenance of Wnt signaling and an increase of Wnt10a, Wnt10b, and Dkk4 expression, are impaired [8] and further placode down-growth does not occur in primary guard hair follicles [36]. In *Map3k7* ^fl/fl^K5-Cre mice, the number of hair germs was significantly reduced at E15.5, indicating that the development of primary guard hair follicles was greatly impaired. Impaired development of primary guard hair follicles at E15.5 can be explained by the absence of NF-κB activity, due to TAK1 deficiency, consistent with a model in which TAK1 is involved in the EdaA1/EdaR/NF-κB pathway [35] (see Fig. 7). The appearance of a few primary hair placodes might be explained by incomplete TAK1 deletion at E15.5.

In contrast, EdaR/NF-κB activity is dispensable for the induction of awl/auchene hair follicles, as seen in *tabby*, *downless*, and *c^IκBαΔN^* mice, even though EdaR/NF-κB-defective, awl/auchene hair follicles subsequently produce abnormal awl-like hair shafts [36], [37]. EdaR/NF-κB independent Wnt/β-catenin signaling is required for this process [8]. Thus, the placodes that became visible in *Map3k7* ^fl/fl^K5-Cre mice at E16.5 are likely to represent placodes of awl/auchene hair follicles. In a recent study, analyses of Eda and EdaR homologue Troy double-mutant mice revealed that, in addition to primary guard hair follicles, awl/auchene hair follicles were defective in these mice [38]. This study suggested that EdaR and Troy redundantly activate an NF-κB independent pathway, via TRAF6, to develop awl/auchene hair follicle placodes. Therefore, it is conceivable that placode development in *Map3k7* ^fl/fl^K5-Cre mice is controlled by this NF-κB- (and TAK1)-independent pathway (Fig. 7).

Besides NF-κB signaling, recent studies indicate the implication of TAK1 in multiple signaling pathways such as MAP kinases and AP1 signaling [1], [39], supporting the alternative possibility that, in addition to the NF-κB signaling pathway, multiple signaling pathways may be also involved in hair follicle regulation downstream to TAK1. Although some TNF receptor family activate JNK pathway in addition to NF-κB, Edar shows only weak activation of JNK/AP-1 pathway [14], [20]. In contrast to Edar, Troy [40] leads to a strong activation of JNK pathway, but weak activation of NF-κB [20], [40]. Therefore, it is possible Troy/TAK1/JNK/AP-1 pathway is involved in hair morphogenesis. However, this point should be further clarified.

An interesting difference was observed between *Map3k7* ^fl/fl^K5-Cre mice and *tabby*, *downless*, and *c^IκBαΔN^* mice. While *tabby*, *downless*, and *c^IκBαΔN^* mice produced abnormal awl-like hair shafts, *Map3k7* ^fl/fl^K5-Cre mice exhibited a prolonged morphogenesis period and did not develop hair shafts, even after skin transplantation onto normal control mice (unpublished, preliminary findings). This suggests that TAK1 deletion also affects the morphogenesis of the secondary hair follicles.

Although numerous molecular players have been identified as powerful regulators of hair follicle cycling, the exact molecular machinery that drives the elusive “hair cycle clock” remains unclear [29], [41], [42], [43]. For example, IGF-1, HGF, glial-derived neurotrophic factor, and VEGF are known to prolong the duration of anagen, while fibroblast growth factor 5, TGFβ1 and TGFβ2, IL-1β, NT-3, estrogen receptor-mediated signaling, and IFN-γ are all known to induce the anagen-catagen transition [29], [41], [43], [44], [45]. The expression of regulatory molecules is controlled by upstream signals, such as those provided by the NF-κB, Wnt/β-catenin, bone morphogenetic protein (BMP), and Shh-Gli pathways [29], [41], [42]. On the basis of our findings, TAK1 should now be considered a part of the molecular machinery of the anagen induction.

In the present study, we have shown that TAK1 deletion severely delays the telogen-anagen transition, although it is not completely suppressed, while deletion of TAK1 in anagen follicles prematurely induces catagen and damages normal hair follicle function. Thus, TAK1 activity is important, but not essential, for anagen initiation and progression, yet is essential for the maintenance of mature anagen follicles, with loss of TAK1 activity resulting in catagen induction. The next challenge is to dissect the upstream and downstream signals of TAK1. Because NF-κB is thought to be a downstream signal of TAK1 in hair morphogenesis, and because strong NF-κB activity is detected in the anagen matrix of pelage follicles of adult mice [37], NF-κB is a major candidate downstream signal of TAK1 in anagen induction. Conversely, EdaR is a plausible candidate upstream signal of TAK1 because EdaA1 and EdaR have already been shown to be involved in hair follicle cycling [9], [21], [22].

After chemical, biological, or physical damage, hair follicles develop abnormalities that are collectively called hair follicle dystrophy [28]. Low follicular damage induces the “dystrophic anagen” response. Severe follicular damage induces the “dystrophic catagen” response, characterized by an immediate anagen termination. In Fig. 6, the hair follicle stage was defined as “dystrophic catagen”. The development of “dystrophic catagen” indicates TAK1-deletion-induced follicular damage is comparatively higher and similar to those of high dose cyclophosphamide-induced alopecia. These suggest that TAK1-deletion-induced dystrophic catagen could be a model of chemotherapy-induced alopecia.

The evidence reported here, that TAK1, a critical mediator of inflammation [1], [30], is also involved in hair morphogenesis and anagen induction.
